# Phenotypes of osteoarthritis-related knee pain and their transition over time: data from the osteoarthritis initiative

**DOI:** 10.1186/s12891-024-07286-4

**Published:** 2024-02-24

**Authors:** Jing Ye, Dongxing Xie, Xiaoxiao Li, Na Lu, Chao Zeng, Guanghua Lei, Jie Wei, Jiatian Li

**Affiliations:** 1 Department of Orthopaedics, Xiangya Hospital, Central South University, Changsha, China; 2 Hunan Key Laboratory of Joint Degeneration and Injury, Xiangya Hospital, Central South University, Changsha, China; 3 Key Laboratory of Aging-related Bone and Joint Diseases Prevention and Treatment, Ministry of Education, Xiangya Hospital, Central South University, Changsha, China; 4 Arthritis Research Canada, Richmond, Canada; 5 National Clinical Research Center for Geriatric Disorders, Xiangya Hospital, Central South University, Changsha, China; 6 Department of Epidemiology and Health Statistics, Xiangya School of Public Health, Central South University, Changsha, China

**Keywords:** Pain, Osteoarthritis, Phenotype, Risk factors

## Abstract

**Background:**

Identification of knee osteoarthritis (OA) pain phenotypes, their transition patterns, and risk factors for worse phenotypes, may guide prognosis and targeted treatment; however, few studies have described them. We aimed to investigate different pain phenotypes, their transition patterns, and potential risk factors for worse pain phenotypes.

**Methods:**

Utilizing data from the Osteoarthritis Initiative (OAI), pain severity was assessed using the Western Ontario and McMaster Universities Osteoarthritis Index (WOMAC) pain subscale. We identified the activity-related pain phenotypes and estimated the transition probabilities of pain phenotypes from baseline to the 24-month using latent transition analysis. We examined the risk factors at baseline with the 24-month pain phenotypes and the transition of pain phenotypes.

**Results:**

In 4796 participants, we identified four distinct knee pain phenotypes at both baseline and 24-month follow-up: no pain, mild pain during activity (Mild P-A), mild pain during both rest and activity (Mild P-R-A), and moderate pain during both rest and activity (Mod P-R-A). 82.9% knees with no pain at baseline stayed the same at 24-month follow-up, 17.1% progressed to worse pain phenotypes. Among “Mild P-A” at baseline, 32.0% converted to no-pain, 12.8% progressed to “Mild P-R-A”, and 53.2% remained. Approximately 46.1% of “Mild P-R-A” and 54.5% of “Mod P-R-A” at baseline experienced remission by 24-month. Female, non-whites, participants with higher depression score, higher body mass index (BMI), higher Kellgren and Lawrence (KL) grade, and knee injury history were more likely to be in the worse pain phenotypes, while participants aged 65 years or older and with higher education were less likely to be in worse pain phenotypes at 24-month follow-up visit. Risk factors for greater transition probability to worse pain phenotypes at 24-month included being female, non-whites, participants with higher depression score, higher BMI, and higher KL grade.

**Conclusions:**

We identified four distinct knee pain phenotypes. While the pain phenotypes remained stable in the majority of knees over 24 months period, substantial proportion of knees switched to different pain phenotypes. Several socio-demographics as well as radiographic lesions at baseline are associated with worse pain phenotypes at 24-month follow-up visit and transition of pain phenotypes.

**Supplementary Information:**

The online version contains supplementary material available at 10.1186/s12891-024-07286-4.

## Introduction

Knee osteoarthritis (OA) is a common joint disorder accompanied by chronic pain. Numerous studies have reported that knee OA accounts for lower extremity disability more than any other disease and causes a formidable societal burden [1–3]. Despite its high prevalence and global impact, no disease-modifying treatments for OA have been approved [4]. Currently, available OA management is mainly palliative and aimed to relieve pain [3, 5]. However, not all people with OA respond similarly to therapy with specific analgesics in clinical practice, making clinical pain management of OA a challenge [6, 7].

Pain experience in people with OA is characterized by a complicated and multifactorial nature [5]. Its presentations can be various because OA pain is likely affected by genetic, mechanical, psychological, and neurological factors [5]. Recently, a strong emphasis has been placed on the identification of OA phenotypes to guide prognosis and targeted treatment [8]. Previous studies have categorized people with knee OA into distinct profiles according to different criteria [9], such as anatomic, biochemical, epidemiologic, or genetic factors [10–13]. Phenotyping of pain was considered as a research priority for the management of OA [14].

There is growing recognition of the importance of distinguishing between pain-on-movement and pain-at-rest and identifying the unique risk factors for each pain phenotype so that more efficient and appropriate prevention and treatment approaches to each pain phenotype can be developed [15, 16]. Pain-on-movement and pain-at-rest are two different manifestations of knee pain in OA. OA related pain is often activity related [17], and a key distinguishing feature of OA-related pain from that of inflammatory arthritis-related pain is that the pain of OA is typically worsened with activity and relieved with rest [18]. Pain-at-rest has been found to occur in individuals with greater OA severity [19]. In general, the intensity of pain experienced during activity among patients with knee OA is often higher than pain experienced during the rest, and the former often occurred earlier in the disease course [20], while the presence of pain-at-rest is often associated with more advanced knee OA severity and less favorable outcomes [21, 22]. Clinical and experimental studies have also reported pain experienced at rest and on movement respond differently to some pharmacologic regimens in people with OA [23, 24].

Although the precise biological mechanisms remain unknown, it has been postulated that pain during activity is a result of both central and peripheral sensitization, but pain during rest is caused by peripheral sensitization [25], indicating that these two symptomatic profiles may represent distinct pain phenotypes in knee OA. Identifying these pain phenotypes and their transitions allows clinicians to consider factors such as the patient’s pain triggers, timing and potential duration of pain episodes, and whether the characteristics of pain may change in the future. These considerations would enable the development of appropriate treatment strategies, selection of suitable treatment durations, and intervals for follow-up, all of which are essential when devising personalized treatment plans.

Recently, several studies have defined the phenotype of knee OA according to pain symptom [14, 26–30]. However, data of pain from most of these studies were collected cross-sectionally [26–30] and focused on pain sensitivity [14, 26, 28]. To date, there is still a lack of longitudinal research focusing on the changes and progression of OA pain phenotypes, as well as the investigation of pain-on-movement and pain-at-rest, the two distinct types of pain experienced by individuals with OA.

To fill in this knowledge gap, we conducted a study to describe different pain phenotypes and their transition patterns over time and to identify the potential risk factors for worse pain phenotypes using data from the Osteoarthritis Initiative (OAI).

## Methods

### Study population

The OAI is a multi-center longitudinal observational study of risk factors for both incident and progressive knee OA. Individuals (*n* = 4796, 41.5% men) between 45 to 79 years old were recruited from four clinical sites: Baltimore MD, Pittsburgh PA, Pawtucket RI, and Columbus OH. Data for each participant were collected at baseline and annual follow-up visit. A detailed description regarding the rationale and approach of the OAI can be found at https://nda.nih.gov/oai/about-oai. In the current analysis, we used data collected from the baseline and 24-month follow-up visit where the assessments of knee pain are publicly available.

### Assessment of pain

The Western Ontario and McMaster Universities Osteoarthritis Index (WOMAC) questionnaire is one of the most commonly used instruments to assess knee pain in persons with or at risk of knee OA [31]. At baseline and each follow-up visit, WOMAC questionnaire was administered to assess pain. The WOMAC pain subscale comprises five items (i.e., pain when walking, pain when climbing or going down stairs, pain when lying in bed, pain when sitting or lying down, and pain when standing). Each item rated from 0 to 4: 0 = none, 1 = mild, 2 = moderate, 3 = severe, 4 = extreme [31]. The items of pain when walking, pain when climbing or going down stairs, and pain when standing were considered as activity items, which reflected to at least mild physical intensity. Pain when lying in bed, pain when sitting or lying down were considered as rest items [16]. In the OAI, the questions for knee-specific pain were asked within the past 7 days.

### Assessment of covariates

Socio-demographic variables (i.e., age, sex, race, education), history of knee injury (defined as knee injured badly enough to limit ability to walk for at least 2 days) were collected at baseline. Depression was assessed by the Center for Epidemiological Studies-Depression (CES-D) score. Participants rated their feelings such as having appetite, feeling depressed, restless, fearful, lonely, happy, sad, hopeful for the future, having crying spells, etc. (20 questions) for the past week from 1 (=rarely or none of the time; < 1 day) to 4 (=most or all of the time; 5–7 days) [32]. Participants were weighed (using a balance beam scale) without shoes or heavy clothes. Height was measured (using a stadiometer) without shoes at baseline clinic examination. Body mass index (BMI) was computed as weight (kg)/height (m)^2^. Kellgren and Lawrence (KL) grade at the tibiofemoral joint was assessed at the central reading center.

### Statistical analysis

**Fig. 1:**
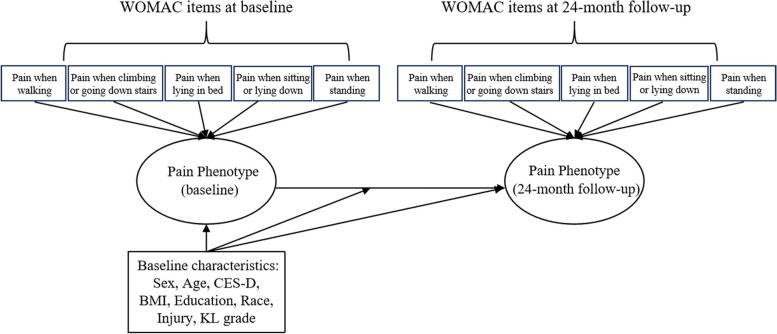
Path diagram for pain phenotype transition from baseline to 24-month follow-up through latent transition model. Five WOMAC items at baseline and 24-month follow-up were used to distinguish different pain phenotypes at baseline and 24-month follow-up, respectively. Baseline characteristics (e.g., sex, age and BMI) were used in the log binomial model and latent transition model for analyzing the factors associated with the 24-month pain phenotype and phenotype transitions. BMI, body mass index; CES-D, Center for Epidemiological Studies-Depression; KL garde, Kellgren and Lawrence grade; WOMAC, Western Ontario and McMaster Universities Osteoarthritis Index

We performed latent transition analysis (LTA) to identify latent pain phenotypes and estimate the transition probability of each pain phenotype from baseline to other pain phenotypes at 24-month follow-up visit. LTA consists of three sets of parameters, including pain phenotype probability at baseline, transition probability of specific pain phenotype from baseline to other pain phenotypes, and 5 item-response probabilities of the WOMAC pain-subscale at baseline and 24-month follow-up visit [33]. Specifically, we first performed latent class analysis (LCA) and LTA to group knees into homogenous phenotypes (i.e., clusters) using WOMAC items, with each cluster composed of knees that share similar observed characteristics (i.e., responses to 5 pain items and its severity) that are distinct from those defining other phenotypes. A specific pain phenotype was identified by the five item-response probabilities (range: 0–1), where a high probability of a particular item indicates that the participants in that phenotype responded high for that item [34]. The model also generates the transition probability among different phenotypes of knee pain. We assessed model fit using the Akaike Information Criterion (AIC), the Bayesian Information Criterion (BIC) and the sample size-adjusted BIC (ABIC). The best-fitting model was identified by considering the lowest AIC, BIC and ABIC values. In addition, the clinical relevance and interpretability of the clusters were also considered during model selection [35]. Figure 1 depicts a path diagram of studying change of one pain phenotype to another from baseline to 24-month follow-up visit using latent transition model. We identified four distinct knee pain phenotypes at both baseline and 24-month follow-up visit based on best-fitting model (Supplement Table 1): “No Pain”, “mild pain during activity” (Mild P-A), “mild pain during both rest and activity” (Mild P-R-A), and “moderate pain during both rest and activity” (Mod P-R-A). Finally, we estimated the transition probabilities of pain phenotypes from baseline to the 24-month follow-up visit using LTA [36]. Log binomial model was performed to explore the relation of baseline predictors, i.e., age, race, sex, education, BMI, CES-D, injury history and KL grade, to the 24-month follow-up visit pain phenotype membership and the accociation between baseline predictors and pain phenotype membership transition from baseline to 24-month follow-up visit were estimated using multivariable regression of latent transition model, respectively.

Latent Class Analysis and Latent transition Analysis were performed using PROC LCA and PROC LTA procedure (version 1.3.2; 2015) in SAS 9.4 (SAS Institute). Log binomial model was performed with lbm package in R 4.3.

## Results

Of the 4796 participants from OAI, 4282 participants (8509 knees, 57.8% women, mean age: 61.2 years) had WOMAC pain score available at both baseline and 24-month follow-up visit. Baseline characteristics according to the pain phenotype membership at baseline were shown in Table 1. Compared with the “No Pain”, “Mild P-A”, and “Mild P-R-A” phenotypes, the “Mod P-R-A” phenotype has a higher proportion of female, non-whites, higher CES-D, higher BMI, lower education level, higher prevalence of history of knee injury, and more severe radiographic knee OA.

**Table 1:** Baseline characteristics according to the baseline pain phenotype membership

Characteristics	No Pain	Mild P-A	Mild P-R-A	Mod P-R-A
Sex (female, %)	55.8	59.4	58.3	69.8
Age, years, mean (SD)	61.4 (9.2)	61.5 (9.1)	60.6 (9.2)	60.6 (8.7)
CES-D, mean (SD)	5.3 (6.0)	6.3 (6.1)	8.1 (7.7)	11.1 (9.1)
BMI, kg/m^2^, mean (SD)	27.7 (4.5)	28.6 (4.6)	29.7 (4.8)	31.5 (5.5)
Education (college of above, %)	88.4	87.1	79.2	65.5
Race (non-whites, %)	12.2	17.3	27.3	51.6
Injury (yes, %)	19.9	30.2	39.2	43.2
Baseline KL				
0	48.3	32.1	26.3	16.9
1	20.1	17.3	15.5	8.5
2	21.9	23.1	27.6	35.3
3	9.1	15.0	21.6	27.8
4	0.6	3.5	8.7	11.5

*Mild P-A* mild pain during activity, *Mild P-R-A* mild pain during both rest and activity, *Mod P-R-A* moderate pain during both rest and activity, *CES-D* Center for Epidemiological Studies-Depression, *BMI* body mass index, *KL* Kellgren and Lawrence

Quantitative variables are shown as mean (SD), and qualitative variables are shown as (%)

**Fig. 2:**
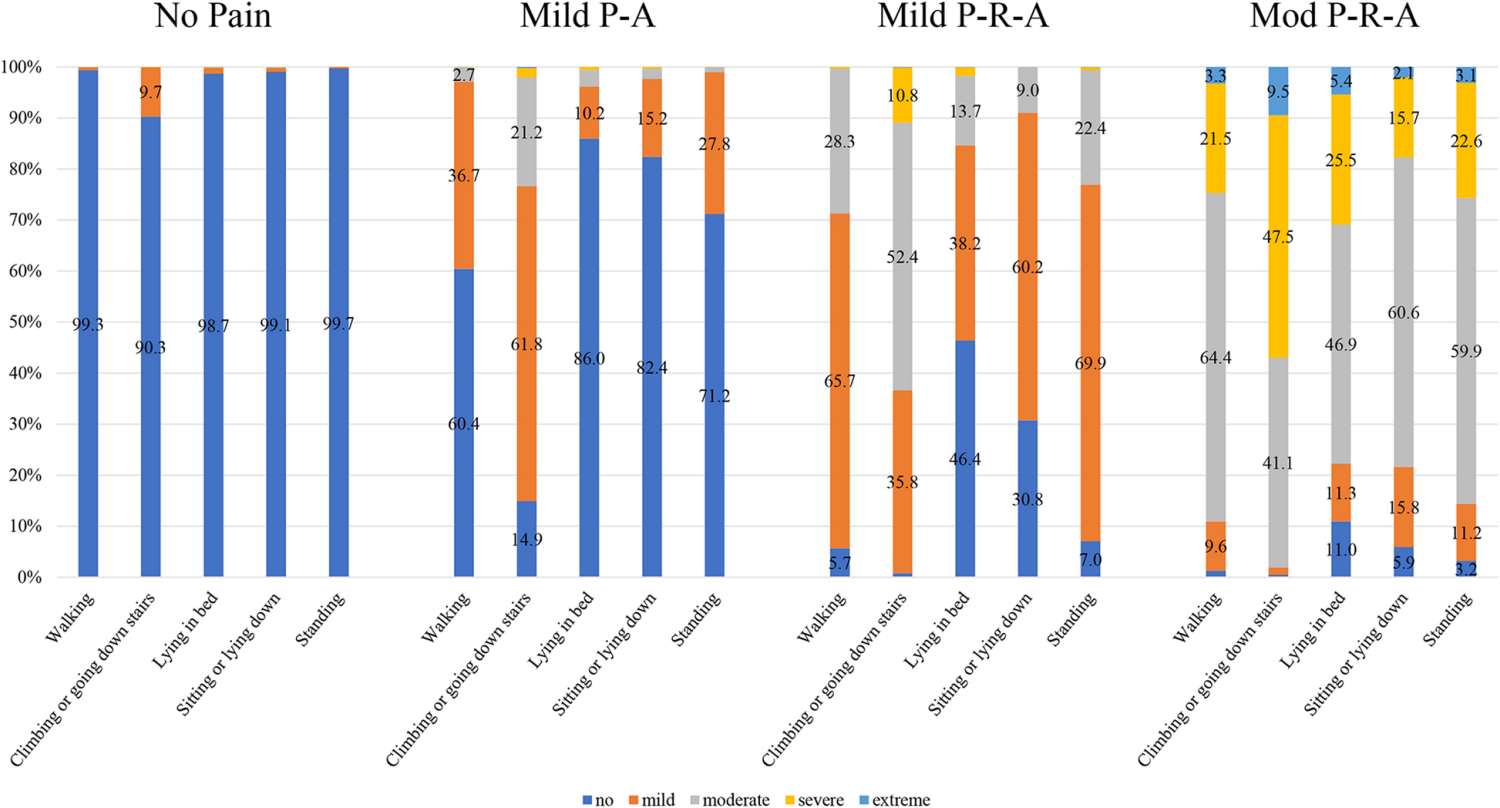
Proportions of individuals in each phenotype for all items among total knees. Mild P-A, mild pain during activity; Mild P-R-A, mild pain during both rest and activity; Mod P-R-A, moderate pain during both rest and activity

The item-response probabilities are showed in Fig. 2. More than 90.0% knees of “No Pain” phenotype responded “no” to all five WOMAC pain items. Among the knees of “Mild P-A” phenotype, 61.8% knees had “mild” pain during climbing or going down stairs, and 36.7% knees had “mild” pain during walking. Of the knees of “Mild P-R-A” phenotype, 65.7% had “mild” pain during walking; 35.8% experienced “mild” and 52.4% had “moderate” pain during climbing or going down stairs; 69.9% had “mild” pain during standing; 60.2% had “mild” pain during sitting or lying down; and 38.2% had “mild” pain when lying in bed. Among the knees of “Mod P-R-A” phenotype, more than 75.0% experienced “moderate” or “severe” or “extreme” to all five WOMAC pain items.

**Fig. 3:**
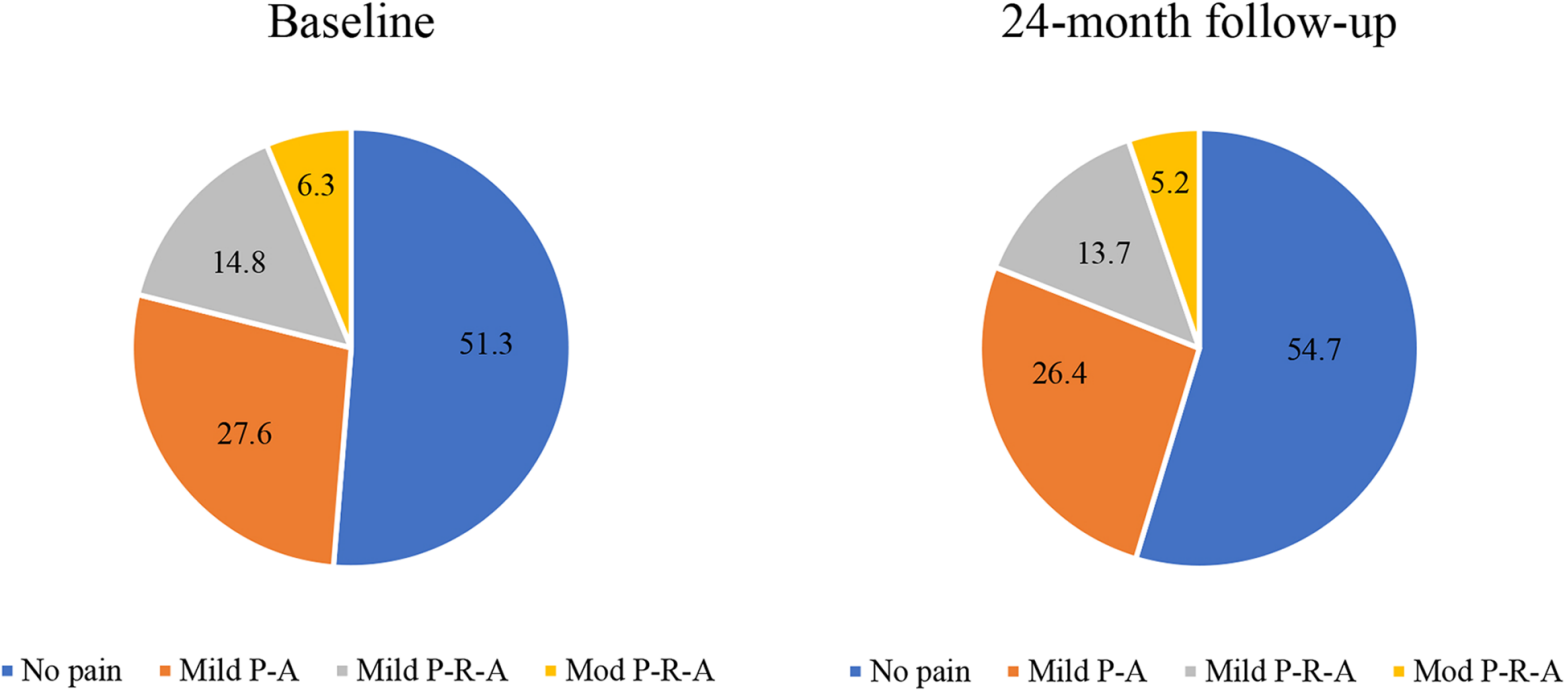
Probabilities of status membership at baseline and 24-month follow-up. Mild P-A, mild pain during activity; Mild P-R-A, mild pain during both rest and activity; Mod P-R-A, moderate pain during both rest and activity

Slightly more than half knees were grouped into “No Pain” phenotype at both baseline and 24-month follow-up visit; approximately one-quarter of knees at baseline (27.6%) and at 24-month follow-up visit (26.4%) belonged to “Mild P-A” phenotype; “Mild P-R-A” phenotype included 14.8% knees at baseline and 13.7% knees at 24-month follow-up visit; about 5% of knees at baseline and at 24-month follow-up visit were classified to “Mod P-R-A” phenotype (Fig. 3).

During the follow-up, a majority of knees of “No Pain” phenotype at baseline stayed in the same phenotype; however, 13.2% of knees developed “Mild P-A” at 24-month follow-up visit. Of the knees of “Mild P-A” phenotype at baseline, 32.0% showed an improvement (i.e., converted to “No Pain”) at 24-month follow-up visit, whereas 14.9% had their pain worsened at 24-month follow-up visit, i.e., progressed to “Mild P-R-A” phenotype or “Mod P-R-A” phenotype. Approximately 46.1% of knees of “Mild P-R-A” at baseline had their pain improved to either “No Pain” or “Mild P-A” at 24-month follow-up visit. Among the knees of “Mod P-R-A” at baseline, 32.3% improved to “Mild P-R-A” and 22.2% improved to other pain phenotypes at 24-month follow-up visit (Table 2). After analyzing the transition probability of the incidence cohort (3284 participants who without symptomatic knee OA, but were at elevated risk of developing symptomatic knee OA) and the progression cohort (1390 participants who were symptomatic knee OA patients) of the OAI separately, the knees of the progression cohort with “No pain” and “Mild P-A” phenotype at baseline showed a higher likelihood of transitioning to more severe phenotypes at 24-month follow-up visit compared to the incidence cohort (23.4% vs. 17.2%, 37.8% vs. 11.9%). Additionally, for knees with the “Mod P-R-A” phenotype at baseline, both the incidence cohort and progression cohort indicate more than half knees improved to other pain phenotypes at 24-month follow-up visit (Supplement Table 2, Supplement Table 3).

**Table 2:** Transition probability of pain phenotype from baseline to 24-month follow-up

Baseline phenotype	24-month follow-up phenotype			
	No Pain	Mild P-A	Mild P-R-A	Mod P-R-A
No Pain	0.829	0.132	0.030	0.009
Mild P-A	0.320	0.531	0.128	0.021
Mild P-R-A	0.174	0.287	0.449	0.090
Mod P-R-A	0.119	0.103	0.323	0.455

*Mild P-A* mild pain during activity, *Mild P-R-A* mild pain during both rest and activity, *Mod P-R-A* moderate pain during both rest and activity

As shown in Table 3, within the “Mild P-A”, “Mild P-R-A”, and “Mod P-R-A” pain phenotype membership, being female and non-whites, participants with higher CES-D score, higher BMI, higher KL grade and having knee injury history at baseline were significantly more likely to be in the worse pain phenotypes than in the “No Pain” phenotype at 24-month follow-up visit. Participants aged 65 years or older and with higher education were significantly less likely to be in worse pain phenotypes at 24-month follow-up visit.

**Table 3:** Associations between baseline characteristics and pain phenotypes at 24-month follow-up

Baseline characteristics	No Pain RR (95% CI)	Mild P-A RR (95% CI)	Mild P-R-A RR (95% CI)	Mod P-R-A RR (95% CI)
Age, year^a^				
< 60	1.00 (reference)	1.00 (reference)	1.00 (reference)	1.00 (reference)
≥ 65	1.00 (reference)	1.01 (0.94, 1.08)	**0.89 (0.80, 0.99)**	**0.68 (0.56, 0.82)**
Gender^b^				
Female	1.00 (reference)	**1.11 (1.04, 1.20)**	**1.16 (1.05, 1.29)**	**1.89 (1.55, 2.31)**
CES-D score	1.00 (reference)	**1.01 (1.00, 1.01)**	**1.02 (1.02, 1.03)**	**1.04 (1.03, 1.14)**
BMI, kg/m^2^				
< 25	1.00 (reference)	1.00 (reference)	1.00 (reference)	1.00 (reference)
25–29.9	1.00 (reference)	1.08 (0.98, 1.19)	**1.43 (1.21, 1.68)**	**2.01 (1.46, 2.75)**
≥ 30	1.00 (reference)	**1.21 (1.10, 1.33)**	**1.81 (1.55, 2.13)**	**1.92 (1.40, 2.64)**
Education				
College or above	1.00 (reference)	0.96 (0.87, 1.06)	**0.83 (0.75, 0.93)**	**0.80 (0.72, 0.88)**
Race				
Whites	1.00 (reference)	1.00 (reference)	1.00 (reference)	1.00 (reference)
Non-whites	1.00 (reference)	1.04 (0.96, 1.14)	**1.22 (1.11, 1.35)**	**2.08 (1.74, 2.49)**
Injury	1.00 (reference)	**1.18 (1.10, 1.27)**	**1.34 (1.22, 1.47)**	**1.30 (1.17, 1.44)**
KL grade				
0	1.00 (reference)	1.00 (reference)	1.00 (reference)	1.00 (reference)
1	1.00 (reference)	**1.28 (1.15, 1.43)**	**1.32 (1.10, 1.58)**	**1.45 (1.03, 2.05)**
2	1.00 (reference)	**1.63 (1.49, 1.79)**	**2.04 (1.76, 2.36)**	**3.08 (2.36, 4.02)**
3	1.00 (reference)	**1.84 (1.65, 2.06)**	**3.00 (2.58, 3.49)**	**3.88 (2.95, 5.10)**
4	1.00 (reference)	**2.63 (2.29, 3.01)**	**4.00 (3.45, 4.65)**	**5.42 (4.18, 7.03)**

*Mild P-A* mild pain during activity, *Mild P-R-A* mild pain during both rest and activity, *Mod P-R-A* moderate pain during both rest and activity, *RR* relative risk, *CI* confidence interval, *CES-D* Center for Epidemiological Studies-Depression, *BMI* body mass index, *KL* Kellgren and Lawrence

^a^Age and ^b^gender were adjusted mutually

The rest factors were adjusted for all variables listed above

The multivariable regression results of the association between baseline predictors and pain phenotype transition were showen in Table 4. In general, female, non-whites, participants with higher CES-D score, higher BMI, and higher KL grade were associated with greater transition probability across time from better pain phenotype to worse pain phenotype. Male, whites, participants without knee injury history and lower KL grade were associated with higher probability from worse pain phenotype to better ones, though the effect estimates were relatively small. Age and education were not associate with the pain phenotype membership transition.

**Table 4:** Association between baseline characteristics and pain phenotype membership transition from baseline to 24-month follow-up

Baseline characteristics	Pain phenotype membership transition			
Female	No Pain OR (95% CI)	Mild P-A OR (95% CI)	Mild P-R-A OR (95% CI)	Mod P-R-A OR (95% CI)
No Pain	1.00 (reference)	**1.06 (1.01, 1.12)**	1.05 (0.99, 1.14)	**1.49 (1.18, 1.63)**
Mild P-A	0.98 (0.95, 1.01)	1.00 (reference)	1.02 (0.99, 1.05)	1.07 (0.99, 1.26)
Mild P-R-A	**0.96 (0.93, 0.99)**	1.00 (0.98, 1.02)	1.00 (reference)	1.03 (0.99, 1.05)
Mod P-R-A	0.97 (0.95, 1.01)	0.98 (0.96, 1.00)	0.98 (0.97, 1.00)	1.00 (reference)
Age				
No Pain	1.00 (reference)	1.00 (1.00, 1.00)	0.99 (0.99, 1.00)	0.99 (0.98, 1.00)
Mild P-A	1.00 (1.00, 1.00)	1.00 (reference)	1.00 (0.99, 1.00)	1.00 (0.99, 1.00)
Mild P-R-A	1.00 (1.00, 1.00)	1.00 (1.00, 1.00)	1.00 (reference)	1.00 (1.00, 1.00)
Mod P-R-A	1.00 (1.00, 1.00)	1.00 (1.00, 1.00)	1.00 (1.00, 1.00)	1.00 (reference)
CES-D score				
No Pain	1.00 (reference)	**1.01 (1.00, 1.01)**	**1.02 (1.01, 1.02)**	**1.03 (1.01, 1.03)**
Mild P-A	1.00 (1.00, 1.00)	1.00 (reference)	**1.01 (1.00, 1.01)**	**1.01 (1.00, 1.01)**
Mild P-R-A	1.00 (0.99, 1.00)	0.99 (0.99, 1.00)	1.00 (reference)	**1.00 (1.00, 1.01)**
Mod P-R-A	0.99 (0.99, 1.00)	1.00 (0.99, 1.00)	1.00 (1.00, 1.00)	1.00 (reference)
25 kg/m^2^ ⩽ BMI < 30 kg/m^2^				
No Pain	1.00 (reference)	1.00 (0.95, 1.07)	**1.35 (1.21, 1.46)**	**1.58 (1.30, 1.78)**
Mild P-A	1.02 (0.97, 1.07)	1.00 (reference)	**1.06 (1.00, 1.09)**	1.16 (0.99, 1.34)
Mild P-R-A	1.01 (0.97, 1.05)	**0.96 (0.94, 0.98)**	1.00 (reference)	1.04 (0.99, 1.07)
Mod P-R-A	**1.02 (1.00, 1.07)**	1.00 (0.97, 1.02)	1.00 (0.98, 1.03)	1.00 (reference)
BMI ⩾ 30 kg/m^2^				
No Pain	1.00 (reference)	**1.08 (1.01, 1.15)**	**1.33 (1.17, 1.47)**	**1.50 (1.12, 1.79)**
Mild P-A	0.99 (0.95, 1.04)	1.00 (reference)	**1.11 (1.05, 1.14)**	1.09 (0.96, 1.21)
Mild P-R-A	**0.93 (0.90, 0.97)**	**0.95 (0.93, 0.99)**	1.00 (reference)	0.98 (0.95, 1.01)
Mod P-R-A	1.00 (0.98, 1.03)	1.00 (0.97, 1.03)	0.99 (0.98, 1.02)	1.00 (reference)
College education or above				
No Pain	1.00 (reference)	1.02 (0.97, 1.09)	1.02 (0.92, 1.13)	1.08 (0.75, 1.49)
Mild P-A	0.97 (0.94, 1.00)	1.00 (reference)	0.99 (0.95, 1.05)	1.01 (0.87, 1.16)
Mild P-R-A	0.96 (0.92, 1.01)	0.99 (0.97, 1.04)	1.00 (reference)	0.99 (0.89, 1.07)
Mod P-R-A	1.01 (0.94, 1.07)	1.01 (0.97, 1.03)	1.04 (0.99, 1.06)	1.00 (reference)
Non-whites				
No Pain	1.00 (reference)	0.96 (0.91, 1.01)	1.14 (0.99, 1.23)	**1.68 (1.40, 2.18)**
Mild P-A	1.06 (0.98, 1.11)	1.00 (reference)	1.02 (0.95, 1.09)	**1.29 (1.09, 1.50)**
Mild P-R-A	**1.08 (1.02, 1.12)**	**1.04 (1.00, 1.09)**	1.00 (reference)	**1.10 (1.02, 1.16)**
Mod P-R-A	**0.96 (0.93, 0.99)**	0.97 (0.93, 1.01)	0.98 (0.95, 1.02)	1.00 (reference)
Injury				
No Pain	1.00 (reference)	**1.08 (1.01, 1.07)**	1.14 (0.96, 1.24)	0.97 (0.78, 1.05)
Mild P-A	1.00 (0.97, 1.03)	1.00 (reference)	1.04 (0.97, 1.08)	0.96 (0.87, 1.16)
Mild P-R-A	0.98 (0.93, 1.01)	0.99 (0.96, 1.02)	1.00 (reference)	1.00 (0.96, 1.05)
Mod P-R-A	0.99 (0.96, 1.02)	**0.96 (0.93, 0.99)**	**0.97 (0.96, 0.99)**	1.00 (reference)
KL grade = 1				
No Pain	1.00 (reference)	**1.12 (1.02, 1.20)**	**1.27 (1.06, 1.40)**	**1.52 (1.06, 2.16)**
Mild P-A	**0.92 (0.90, 0.98)**	1.00 (reference)	0.98 (0.91, 1.02)	**1.24 (1.04, 1.45)**
Mild P-R-A	1.01 (0.98, 1.04)	1.00 (0.98, 1.04)	1.00 (reference)	**0.95 (0.88, 0.98)**
Mod P-R-A	**0.96 (0.92, 0.99)**	0.99 (0.97, 1.01)	0.98 (0.97, 1.00)	1.00 (reference)
KL grade = 2				
No Pain	1.00 (reference)	**1.24 (1.14, 1.33)**	**1.34 (1.19, 1.47)**	**1.38 (1.03, 1.60)**
Mild P-A	**0.87 (0.85, 0.91)**	1.00 (reference)	1.02 (0.97, 1.08)	**1.18 (1.05, 1.25)**
Mild P-R-A	**0.92 (0.90, 0.99)**	0.94 (0.93, 1.00)	1.00 (reference)	1.04 (0.98, 1.08)
Mod P-R-A	0.98 (0.94, 1.02)	1.01 (0.98, 1.02)	1.01 (0.98, 1.04)	1.00 (reference)
KL grade = 3				
No Pain	1.00 (reference)	**1.41 (1.28, 1.56)**	**1.62 (1.43, 1.81)**	**1.82 (1.29, 2.22)**
Mild P-A	**0.84 (0.82, 0.90)**	1.00 (reference)	**1.20 (1.13, 1.30)**	**1.50 (1.20, 1.73)**
Mild P-R-A	**0.88 (0.85, 0.92)**	**0.90 (0.89, 0.94)**	1.00 (reference)	1.03 (0.98, 1.09)
Mod P-R-A	**0.89 (0.85, 0.92)**	**0.93 (0.88, 0.95)**	0.98 (0.93, 1.02)	1.00 (reference)
KL grade = 4				
No Pain	1.00 (reference)	**1.35 (1.21, 1.49)**	**1.19 (1.07, 1.37)**	**1.45 (1.09, 1.76)**
Mild P-A	**0.81 (0.75, 0.89)**	1.00 (reference)	1.06 (0.91, 1.17)	1.06 (0.83, 1.28)
Mild P-R-A	**0.75 (0.70, 0.83)**	**0.86 (0.80, 0.93)**	1.00 (reference)	0.90 (0.84, 1.04)
Mod P-R-A	**0.90 (0.80, 0.96)**	1.02 (0.93, 1.17)	0.94 (0.83, 1.01)	1.00 (reference)

*Mild P-A* mild pain during activity, *Mild P-R-A* mild pain during both rest and activity, *Mod P-R-A* moderate pain during both rest and activity, *OR* odds ratio, *CI* confidence interval, *CES-D* Center for Epidemiological Studies-Depression, *BMI* body mass index, *KL* Kellgren and Lawrence

Baseline characteristics listed above were used as covariates in the multivariable regression

## Discussion

Using data collected from OAI we identified four potential knee pain phenotypes based on responses to the WOMAC pain subscale at both baseline and 24-month follow-up visit. While the pain phenotypes remained stable in majority of knees over time, there was substantial transition of pain phenotypes over 24-month period. In general, female, non-whites, participants with higher CES-D, higher BMI, higher KL grade and having knee injury history were associated with worse pain phenotypes. These findings may have implications for identification of pain phenotype-specific risk factors and development of preventive and treatment strategies for potential pain phenotypes.

### Comparison with previous studies

In contrast to previous studies that defined OA-related pain phenotype mainly based on pain sensitivity response or psychological factors [26–28]. We characterized pain phenotype based on pain-on-movement and pain-at-rest, proposed four distinctive activity-related knee pain phenotypes in this study, reflecting the different pain severity at commonly engaged daily activities. Identification of these two phenotypes holds significant clinical relevance for guiding precision treatment strategies. Moreover, the longitudinal study design allows us to further observe transitions in pain phenotypes among OA patients, which is of substantial significance for predicting prognosis and making corresponding adjustments to treatment strategies. Previous studies have found that most structural lesions in knee OA are either stable or gradually and consistently worsen over time [37, 38]. In our study, we also observed that activity-related pain phenotypes in many individuals were stable over 24-month period. However, pain phenotypes transition, either improvement or worsening, did occur in a substantial proportion of patients, especially those knees with mild or moderate pain during both rest and activity.

To date, there are a paucity of evidence on potential predictors of pain phenotypes because most studies of association of risk factors and knee pain phenotypes were based on cross-sectional design. The present study demonstrated that female, non-whites, participants with higher CES-D, higher BMI, higher KL grade and history of knee injury were significant predictors of worse pain phenotypes at 24-month follow-up visit. Overall, our findings of baseline factors predicting a worsening of pain are similar to previous studies of risk factors for progression of knee pain [39–41]. However, earlier studies primarily emphasized pain intensity, our focus is on changes in pain phenotypes. For clinicians, taking into account a patient’s potential pain patterns and their transition over time is essential when devising personalized treatment plans. Although it has been asserted that there is a poor correlation between structural changes and pain levels in OA [42], we have found some evidence that clinicians should be aware that those who are female, non-whites, participants with higher CES-D score, higher BMI, higher KL grade and having knee injury history appear to have an increased risk of worsening knee OA related pain and may benefit from earlier intervention. Previous studies have found older adults, particularly female and non-whites may be particularly vulnerable developing persistent knee pain potentially related to genetics and/or sociocultural influences, including chronic stress [43, 44]. Also, one study suggested a pain/mental health cycle, where pain leads to depression and fatigue which in turn leads to worsening of pain and function [45].

Remaining overweight increased the risk of knee pain [46], likely because obesity can lead to a loss of muscle mass and strength, as well as fat accumulation, resulting in pressure on the knee joint [47]. Other studies have suggested links between radiographic characteristics and disease progression, with the presence of inflammation increasing the risk of symptomatic progression [48], there is also a growing evidence demonstrating a direct link between knee injury and the subsequent development of OA of the knee [49].

### Strengths and limitations

There are several strengths in our study. Firstly, the present study is the first phenotyping analysis focusing on knee pain-on-movement and pain-at-rest based on a valid instrument, i.e., WOMAC pain subscale. The pain phenotypes identified in our study reflected different pain clusters according to pain occurred when subjects engaged in common daily activities and its severity. In addition, these pain phenotypes showed a relatively high transition probability over a relatively short period. Thus, it provides the investigators another useful pain outcome in future observational studies. Secondly, we used a latent transition model, a novel approach, which is useful to define pain phenotype and to assess the transition probability of the phenotypes. It offers a straightforward classification of participants into mutually exclusive pain patterns, enabling the estimation of transition probabilities, the examination of covariates that elucidate transitions over time, and facilitates comparisons across multiple groups. This method, an agnostic data-driven model-based approach, is less subjective in cluster formation and likely generate more clinically meaningful phenotypes. Several limitations in our study should also be acknowledged. Because participants in OAI consist of those with or at high risk of knee OA, generalizability of our findings, i.e., phenotypes of pain and their transition probability, to other populations should be cautious. Furthermore, the WOMAC pain subscale comprises five distinct items, each with its unique significance. The current “pain-on-movement” and “pain-at-rest” phenotypes were derived through a classification analysis of the five items within the WOMAC pain subscale. Further research and practical application are needed to thoroughly assess the clinical implications of the current phenotypes. In addition, in the current study, we only followed up participants for 24 months, long-term studies are needed to understand the natural history of pain for knee OA.

### Clinical implications

As mentioned above, the distinction between pain-on-movement and pain-at-rest may be associated with different underlying mechanisms, differential treatment responses and thus may have important relevance for the development of new pain treatments. However, in clinical care and clinical research settings, distinguishing between pain-on-movement and pain-at-rest has been limited [50]. Our analysis suggested that the pain phenotypes based on pain-on-movement and pain-at-rest are recognizable and distinguishable. Our study illustrated a new approach to defining pain phenotypes in knee OA and has identified potential factors associated with changes in pain phenotypes. These findings may help understand mechanisms of knee pain, empowering clinicians to customize their focus to individual pain patterns and anticipate potential future variations in pain. This, in turn, can facilitate the development of more personalized and precisely targeted treatment strategies. For instance, doctors may tailor exercise and lifestyle recommendations based on different pain phenotypes and triggering factors, as well as adjust the timing and duration of pain medication usage, which may have the potential to reduce analgesic use frequency and minimize adverse drug reactions in some patients.

## Conclusion

We identified four potential knee pain phenotypes based on responses to the WOMAC pain questionnaire. While the pain phenotypes remained stable in the majority of knees over 24 months period, substantial proportion of knees switched to different pain phenotypes. Several socio-demographics and radiographic lesions at baseline are associated with worse pain phenotypes at 24-month follow-up visit and transition of pain phenotypes.

### Supplementary Information

**Supplementary Materials 1.**

## Data Availability

The datasets used and/or analyzed during the current study are available from the corresponding author upon reasonable request.

## Abbreviations

ABIC: Sample size-adjusted BIC
AIC: Akaike information criterion
BMI: Body mass index
BIC: Bayesian information criterion
CES-D: Center for epidemiological studies-depression
KL: Kellgren and Lawrence
LTA: Latent transition analysis
LCA: Latent class analysis
Mild P-A: Mild pain during activity
Mild P-R-A: Mild pain during both rest and activity
Mod P-R-A: Moderate pain during both rest and activity
OA: Osteoarthritis
OAI: Osteoarthritis initiative
WOMAC: Western Ontario and McMaster Universities Osteoarthritis Index
