# Supplementary material for: Phenotypes of osteoarthritis-related knee pain and their transition over time: data from the osteoarthritis initiative
Source: BMC Musculoskelet Disord. 2024 Feb 24;25:173. doi: 10.1186/s12891-024-07286-4 (PMC10893610; doi:10.1186/s12891-024-07286-4)
Supplement: Supplementary file 1 — Supplementary Materials 1. [file 12891_2024_7286_MOESM1_ESM.docx]

**Supplement table 1.** **Development of optimal model of pain phenotypes using latent class analysis**

| Number of class | AIC | BIC | ABIC | Entropy | Smallest  sample size (%)* |
| --- | --- | --- | --- | --- | --- |
| Baseline |  |  |  |  |  |
| 3 | 3505.55 | 3942.58 | 3745.55 | 0.88 | 10.02 |
| 4 | 2798.20 | 3383.26 | 3199.50 | 0.88 | 6.50 |
| 5 | 2277.00 | 3010.08 | 2679.59 | 0.86 | 4.76 |
| 24-month |  |  |  |  |  |
| 3 | 3504.28 | 3941.31 | 3744.29 | 0.89 | 8.54 |
| 4 | 2705.99 | 3291.05 | 3027.29 | 0.86 | 5.47 |
| 5 | 2219.69 | 2952.77 | 2622.28 | 0.85 | 4.03 |

AIC: Akaike Information Criterion; BIC: Bayesian Information Criterion; ABIC: adjusted BIC.

*Smallest sample size at baseline and 24-month were calculated by latent transition analysis adjusting for the following confounders: sex, age, body mass index, race, education, injury, Center for Epidemiological Studies-Depression score, Kellgren and Lawrence grade.

**Supplement table 2. Transition probability of pain phenotype from baseline to 24-month follow-up for the incidence cohort from the Osteoarthritis Initiative**

| Baseline phenotype | 24-month follow-up phenotype | | | |
| --- | --- | --- | --- | --- |
|  | No Pain | Mild P-A | Mild P-R-A | Mod P-R-A |
| No Pain | 0.828 | 0.129 | 0.032 | 0.011 |
| Mild P-A | 0.373 | 0.508 | 0.096 | 0.024 |
| Mild P-R-A | 0.221 | 0.315 | 0.393 | 0.071 |
| Mod P-R-A | 0.186 | 0.102 | 0.307 | 0.405 |

Mild P-A: mild pain during activity; Mild P-R-A: mild pain during both rest and activity; Mod P-R-A: moderate pain during both rest and activity.

**Supplement table 3. Transition probability of pain phenotype from baseline to 24-month follow-up for the progression cohort from the Osteoarthritis Initiative**

| Baseline phenotype | 24-month follow-up phenotype | | | |
| --- | --- | --- | --- | --- |
|  | No Pain | Mild P-A | Mild P-R-A | Mod P-R-A |
| No Pain | 0.766 | 0.034 | 0.189 | 0.011 |
| Mild P-A | 0.151 | 0.472 | 0.281 | 0.097 |
| Mild P-R-A | 0.278 | 0.154 | 0.557 | 0.012 |
| Mod P-R-A | 0.096 | 0.313 | 0.151 | 0.440 |

Mild P-A: mild pain during activity; Mild P-R-A: mild pain during both rest and activity; Mod P-R-A: moderate pain during both rest and activity.
